# Assessing the feasibility of ChatGPT-4o and Claude 3-Opus in thyroid nodule classification based on ultrasound images

**DOI:** 10.1007/s12020-024-04066-x

**Published:** 2024-10-11

**Authors:** Ziman Chen, Nonhlanhla Chambara, Chaoqun Wu, Xina Lo, Shirley Yuk Wah Liu, Simon Takadiyi Gunda, Xinyang Han, Jingguo Qu, Fei Chen, Michael Tin Cheung Ying

**Affiliations:** 1 Department of Health Technology and Informatics, The Hong Kong Polytechnic University, Kowloon, Hong Kong China; 2 School of Healthcare Sciences, Cardiff University, Cardiff, UK; 3 Department of Ultrasound, The First Affiliated Hospital of Wenzhou Medical University, Wenzhou, China; 4 Department of Surgery, North District Hospital, Sheung Shui, New Territories, Hong Kong China; 5 Department of Surgery, The Chinese University of Hong Kong, Prince of Wales Hospital, Shatin, New Territories, Hong Kong China; 6 Department of Ultrasound, The Fifth Affiliated Hospital of Sun Yat-sen University, Zhuhai, China

**Keywords:** Large language model, Thyroid cancer, Ultrasound, Diagnostic accuracy, Artificial intelligence

## Abstract

**Purpose:**

Large language models (LLMs) are pivotal in artificial intelligence, demonstrating advanced capabilities in natural language understanding and multimodal interactions, with significant potential in medical applications. This study explores the feasibility and efficacy of LLMs, specifically ChatGPT-4o and Claude 3-Opus, in classifying thyroid nodules using ultrasound images.

**Methods:**

This study included 112 patients with a total of 116 thyroid nodules, comprising 75 benign and 41 malignant cases. Ultrasound images of these nodules were analyzed using ChatGPT-4o and Claude 3-Opus to diagnose the benign or malignant nature of the nodules. An independent evaluation by a junior radiologist was also conducted. Diagnostic performance was assessed using *Cohen’s Kappa* and receiver operating characteristic (ROC) curve analysis, referencing pathological diagnoses.

**Results:**

ChatGPT-4o demonstrated poor agreement with pathological results (*Kappa* = 0.116), while Claude 3-Opus showed even lower agreement (*Kappa* = 0.034). The junior radiologist exhibited moderate agreement (*Kappa* = 0.450). ChatGPT-4o achieved an area under the ROC curve (AUC) of 57.0% (95% CI: 48.6–65.5%), slightly outperforming Claude 3-Opus (AUC of 52.0%, 95% CI: 43.2–60.9%). In contrast, the junior radiologist achieved a significantly higher AUC of 72.4% (95% CI: 63.7–81.1%). The unnecessary biopsy rates were 41.4% for ChatGPT-4o, 43.1% for Claude 3-Opus, and 12.1% for the junior radiologist.

**Conclusion:**

While LLMs such as ChatGPT-4o and Claude 3-Opus show promise for future applications in medical imaging, their current use in clinical diagnostics should be approached cautiously due to their limited accuracy.

## Introduction

The widespread use of high-resolution ultrasound technology and increased public health awareness have significantly boosted the detection rates of thyroid nodules [1]. Although the majority of these nodules are benign, accurate differentiation between benign and malignant cases is critical for making informed clinical decisions and ensuring timely and appropriate treatment for malignant nodules [2]. Currently, fine-needle aspiration biopsy (FNAB) and surgical pathology are the gold standards for diagnosing thyroid nodules. While these methods offer high diagnostic accuracy, they are invasive and can cause discomfort and complications for patients [3]. Ultrasound imaging is the primary non-invasive method for evaluating thyroid nodules, offering a safer alternative. However, its diagnostic accuracy heavily depends on the radiologist’s expertise, resulting in variability in clinical outcomes [4].

Large language models (LLMs) have emerged as a transformative force in artificial intelligence (AI), with advanced capabilities in natural language understanding, logical reasoning, and multimodal interactions [5]. Since the introduction of OpenAI’s ChatGPT-3.5 in November 2022, generative AI has rapidly gained prominence [6]. The subsequent release of more sophisticated models, such as ChatGPT-4.0 in 2023, which integrate text, voice, and image processing, has further demonstrated the profound potential of these technologies. Models like Claude 3-Opus and ChatGPT-4o exemplify the rapid evolution of AI, showcasing increasingly human-like cognitive functions. The expanding application of language comprehension AI across various sectors highlights its significant potential for societal advancement [7].

Recent studies have explored the use of LLMs in various medical fields, including medical education [8], clinical diagnosis [9], and healthcare quality management [10], yielding promising results such as improved learning, diagnostic support, and operational efficiency. In the context of thyroid nodule management, recent research has primarily focused on text-based analysis, using LLMs to process clinical reports or ultrasound descriptions to assist in diagnosis [11, 12]. However, with the growing capability of LLMs to handle visual data, there is significant potential for these models to aid in direct medical image analysis [13]. The application of LLMs to the task of distinguishing between benign and malignant thyroid nodules from ultrasound images represents an exciting opportunity to further explore their role in medical imaging.

This study aims to evaluate the potential of LLMs, including Claude 3-Opus and ChatGPT-4o, in distinguishing between benign and malignant thyroid nodules using ultrasound images. By comparing the diagnostic performance of LLMs with that of radiologist, we seek to assess the feasibility of applying LLMs in processing and analyzing medical images, thereby exploring their potential clinical applications.

## Materials and methods

### Ethical statement

This study is a cross-sectional clinical research project approved by the institutional ethics committee of The Hong Kong Polytechnic University and conducted in accordance with the Declaration of Helsinki. Written informed consent was obtained from all patients prior to their participation in the study.

### Study population

The cases included in this study for analysis were derived from a prospectively and consecutively enrolled cohort at our institution between May 2019 and August 2021. All participants underwent thyroid nodule ultrasound examinations, followed by either preoperative FNAC, postoperative histopathological evaluation, or both. The inclusion criteria were: (1) patients aged 18 years or older; (2) patients who underwent thyroid ultrasound examination prior to thyroid nodule FNAB or thyroid surgery; and (3) patients with a definitive pathological diagnosis from FNAB cytology and/or surgical biopsy. The exclusion criteria were: (1) thyroid nodule images with poor quality, defined as those affected by motion artifacts that significantly degraded image clarity or cases where multiple nodules in a single lobe were so closely adjacent that effective segmentation was not possible, making them unsuitable or unfeasible for analysis; and (2) patients with a history of thyroid surgery or medical treatment for thyroid nodules.

**Fig. 1:**
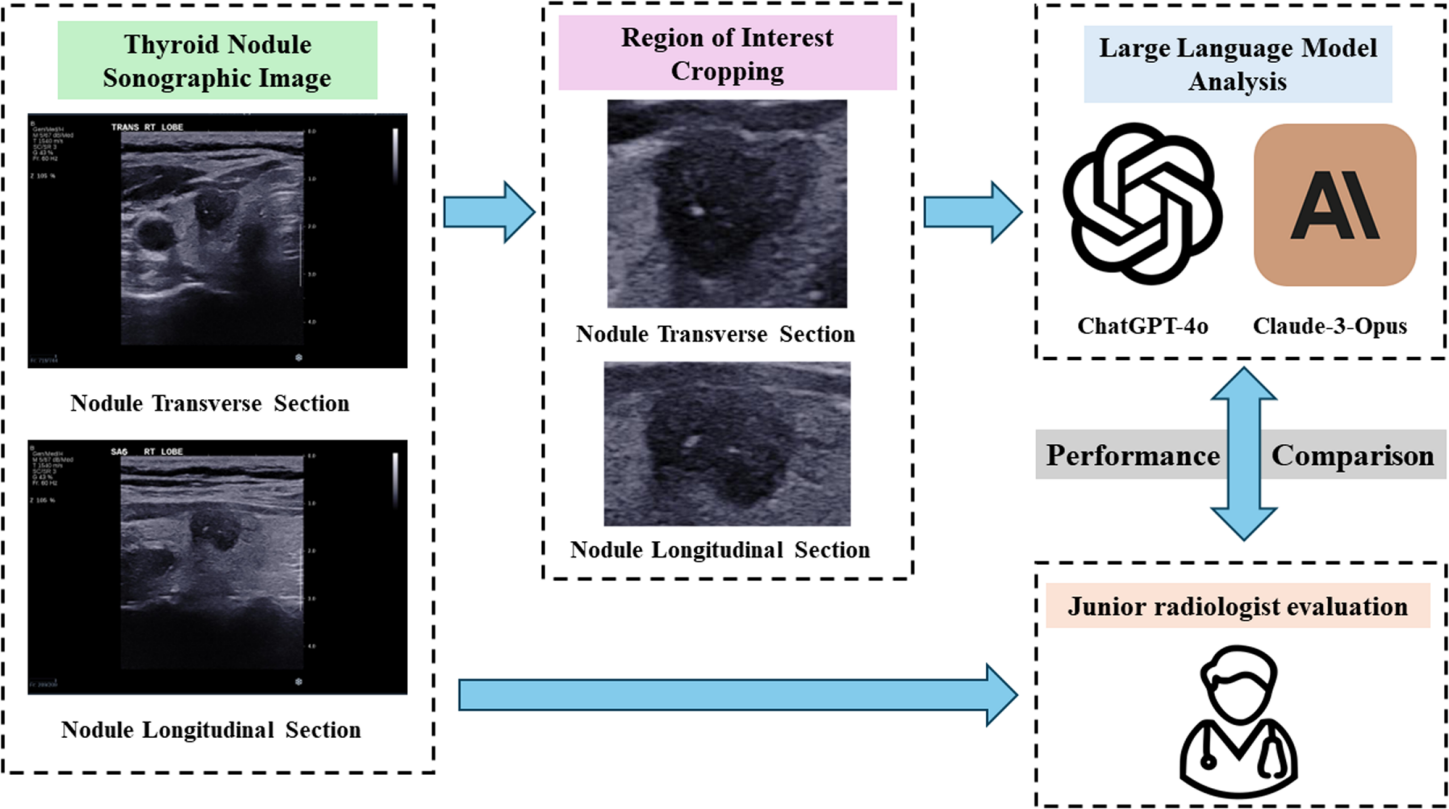
Study Workflow for Comparing Large Language Models and a Junior Radiologist in Thyroid Nodule Classification. This flowchart outlines the study design comparing ChatGPT-4o, Claude 3-Opus, and a junior radiologist in distinguishing between benign and malignant thyroid nodules using ultrasound images. It includes steps such as obtaining sonographic images, cropping regions of interest, and analyzing these images using large language models, followed by performance comparison with a junior radiologist

The overall study design is illustrated in Fig. 1.

### Ultrasound examination

All thyroid ultrasound examinations were conducted by a single sonographer with over three years of experience using the Aixplorer Ultrasound imaging system (SuperSonic Imagine, Aix-en-Provence, France) equipped with a linear array probe (SL15-4, 4–15 MHz). Transverse and longitudinal ultrasound images of the thyroid nodules were stored for analysis. All images used for analysis in this study were the original images. No measurements or markings were included on the images.

### Large language models analysis

**Fig. 2:**
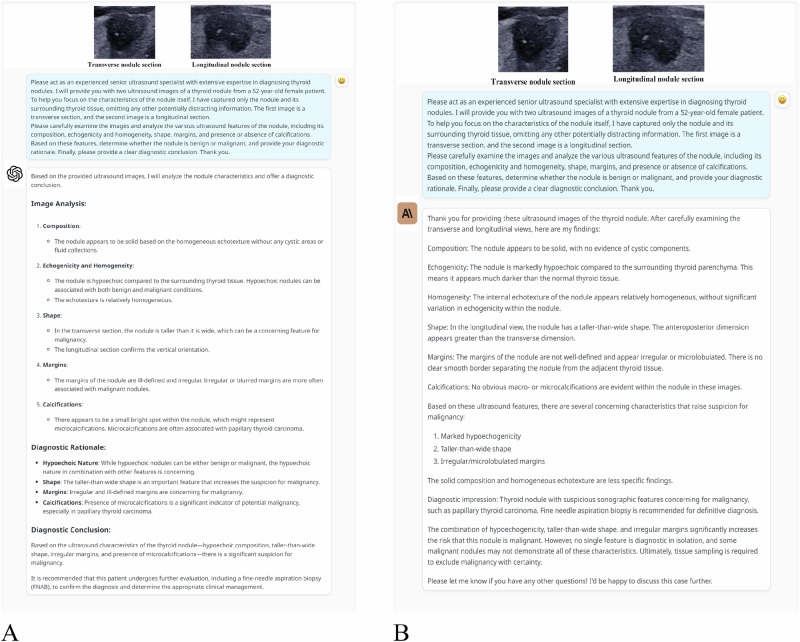
Dialogue-Based Interaction for Thyroid Nodule Ultrasound Image Analysis Using Large Language Models. Input thyroid nodule ultrasound images and prompts into ChatGPT-4o (**A**) and Claude 3-Opus (**B**) to distinguish between benign and malignant thyroid nodules

Two LLMs, ChatGPT-4o (OpenAI, San Francisco, CA, USA) and Claude 3-Opus (Anthropic, San Francisco, CA, USA), were employed for analysis in this study. ChatGPT-4o, developed by OpenAI, was utilized in its most recent version available during the study period, with its training databases updated until October 2023. Similarly, Claude 3-Opus, developed by Anthropic, was used in its most recent version available, with training databases updated until August 2023. Both models were accessed through their respective application programming interface (API) services to ensure consistent and reproducible interaction parameters. The thyroid ultrasound images used in this study originated from our private database, which is not accessible online, thus preventing the LLMs from utilizing these images during pre-training—a process where models are initially trained on large datasets to learn general patterns that can later be applied to specific tasks, such as image classification. For each thyroid nodule, two ultrasound images were used: one transverse and one longitudinal section. These two images were consistently included for every nodule to ensure comprehensive analysis from different angles. The images were meticulously cropped to remove irrelevant details and unrelated anatomical structures, preserving only the nodules and the surrounding thyroid tissue. These prepared images, along with the patient’s age and gender, were then input into the LLMs for analysis. The input prompt was as follows: *“Please act as an experienced senior ultrasound specialist with extensive expertise in diagnosing thyroid nodules. I will provide you with two ultrasound images of a thyroid nodule from a XX-year-old XX (gender) patient. To help you focus on the characteristics of the nodule itself, I have captured only the nodule and its surrounding thyroid tissue, omitting any other potentially distracting information. The first image is a transverse section, and the second image is a longitudinal section. Please carefully examine the images and analyze the various ultrasound features of the nodule, including its composition, echogenicity and homogeneity, shape, margins, and presence or absence of calcifications. Based on these features, determine whether the nodule is benign or malignant, and provide your diagnostic rationale. Finally, please provide a clear diagnostic conclusion. Thank you.”* The LLMs analyzed the ultrasound images and provided diagnostic conclusions regarding the benign or malignant nature of the nodules (Fig. 2). The research analyses using ChatGPT-4o were conducted from June 3 to June 9, 2024, while the analyses using Claude 3-Opus were carried out from June 10 to June 16, 2024. All analyses by the LLMs were performed by an independent operator. With each new analysis, a new chat session was initiated, and records from the previous session were cleared to ensure confidentiality and accuracy.

### Junior radiologist evaluation

Thyroid nodule ultrasound images and patient information (age, gender) were also provided to a junior radiologist with two years of experience for an independent assessment. The radiologist conducted a detailed assessment of the nodule’s characteristics according to the ACR TI-RADS guidelines, including its composition, echogenicity, shape, margins, and echogenic foci. Based on the evaluation of these specific categories, the radiologist provided a final diagnostic conclusion regarding the benign or malignant nature of the nodule.

### Statistical analysis

Data analysis was performed using SPSS 26.0 software (SPSS Inc., Chicago, IL, USA) and R statistical software (version 4.2.0; http://www.R-project.org). Categorical data were expressed as frequencies (percentages), and continuous data as means ± standard deviations. Pathological diagnosis served as the gold standard. *Chi-square* tests were employed to assess the diagnostic capabilities of the LLMs and the junior radiologist in distinguishing between benign and malignant nodules. *Cohen’s Kappa* consistency analysis was used to evaluate the agreement between the LLMs, the junior radiologist, and the pathological diagnosis, with *Kappa* values interpreted as follows: 0–0.2 (poor agreement), 0.2–0.4 (fair agreement), 0.4–0.6 (moderate agreement), 0.6–0.8 (substantial agreement), and 0.8–1.0 (almost perfect agreement). Receiver operating characteristic (ROC) curve analysis was used to assess diagnostic performance, calculating the area under the ROC curve (AUC), sensitivity, specificity, and accuracy. The comparison of AUCs was conducted using the *DeLong* test. The unnecessary biopsy rate, defined as the proportion of misdiagnosed benign nodules among the total biopsy-required nodules, was also calculated. A two-sided *P* value of less than 0.05 was considered statistically significant.

## Results

### Patient and nodule characteristics

A total of 112 patients, encompassing 116 thyroid nodules, were included in this study. Of these, 9 patients (8.0%) underwent only surgical pathology without FNAC, 10 patients (8.9%) had FNAC without surgical pathology, and 93 patients (83.1%) underwent both. The patient cohort consisted of 19 males and 93 females, with a mean age of 53.79 ± 12.14 years. Among the nodules, 75 (64.7%) were benign with an average size of 2.53 ± 1.24 cm, while 41 (35.3%) were malignant with an average size of 1.97 ± 1.32 cm. Detailed baseline characteristics are presented in Table 1.

**Table 1:** Baseline characteristics of patients and thyroid nodules

Characteristic	Total	Benign	Malignant
Patients	112	75	37
Sex (Male/Female)	19/93	12/63	7/30
Age (years)	53.79 ± 12.14	53.32 ± 12.02	54.73 ± 12.48
Nodules	116	75 (64.7)	41 (35.3)
Nodule size (cm)	2.33 ± 1.29	2.53 ± 1.24	1.97 ± 1.32

Categorical variables are presented as *n* (%) and continuous variables as mean ± standard deviation.

### Consistency analysis with pathological results

ChatGPT-4o demonstrated poor agreement with pathological results, reflected by a *Kappa* value of 0.116 (*P* = 0.118). Claude 3-Opus showed even lower agreement, with a *Kappa* value of 0.034 (*P* = 0.653). The *P* values for both models were greater than 0.05, indicating that neither ChatGPT-4o nor Claude 3-Opus could effectively distinguish between benign and malignant nodules. In contrast, the junior radiologist exhibited moderate agreement with a *Kappa* value of 0.450 (*P* < 0.001), indicating statistically significant consistency with pathological diagnoses (Table 2).

**Table 2:** Consistency analysis between diagnostic approaches and pathological results

Index		Pathological result		*Cohen’s Kappa* value	*χ*^*2*^	*P* value
		Benign	Malignant			
ChatGPT-4o	Benign	27	9	0.116	2.444	0.118
	Malignant	48	32			
Claude-3-Opus	Benign	25	12	0.034	0.202	0.653
	Malignant	50	29			
Junior radiologist	Benign	61	15	**0.450**	23.495	< 0.001
	Malignant	14	26			

The bold value signifies the highest diagnostic performance in this metric

### Diagnostic performance comparison: LLMs vs. junior radiologist

**Fig. 3:**
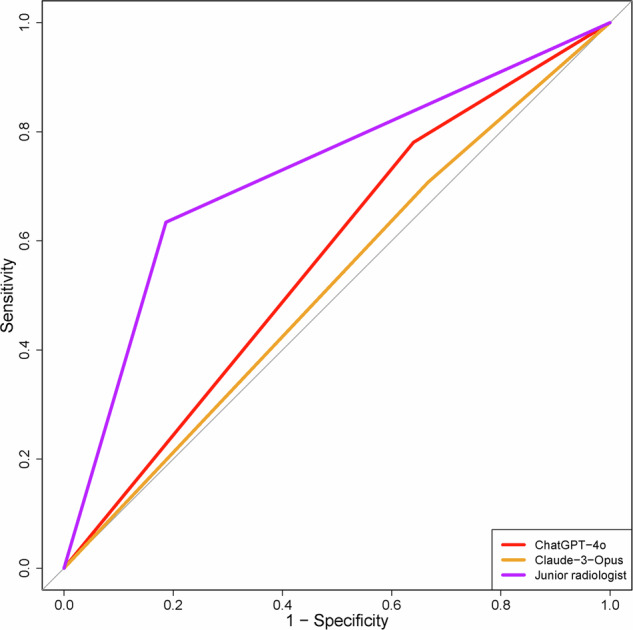
Comparative Performance of Large Language Models and a Junior Radiologist in Thyroid Nodule Classification Using ROC Curve

**Fig. 4:**
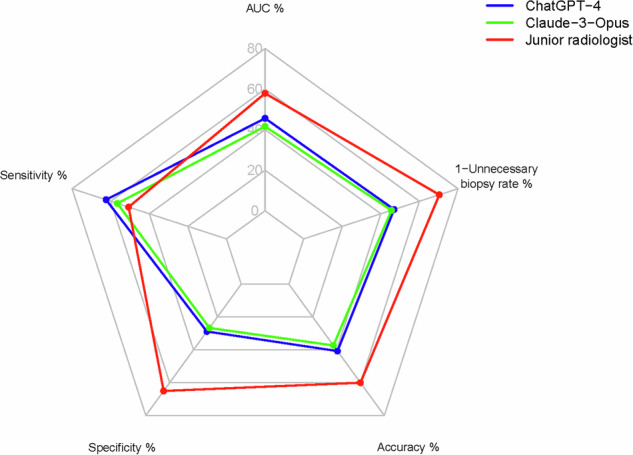
Radar Chart Comparing Key Performance Metrics of ChatGPT-4o, Claude 3-Opus, and a Junior Radiologist in Thyroid Nodule Classification

When comparing diagnostic performances, ChatGPT-4o achieved an AUC of 57.0% (95% CI: 48.6–65.5%), slightly outperforming Claude 3-Opus, which had an AUC of 52.0% (95% CI: 43.2–60.9%). However, the difference between their performances was not statistically significant (*P* = 0.393). Both LLMs exhibited significantly lower diagnostic performance compared to the junior radiologist, who achieved an AUC of 72.4% (95% CI: 63.7–81.1%) (vs. ChatGPT-4o, *P* = 0.008; vs. Claude 3-Opus, *P* = 0.002). The junior radiologist also demonstrated superior accuracy (75.0%, 95% CI: 66.1–82.6%) and specificity (81.3%, 95% CI: 70.7–89.4%) compared to the LLMs, although ChatGPT-4o exhibited the highest sensitivity (78.0%, 95% CI: 62.4–89.4%) (Table 3, Figs. 3, 4).

**Table 3:** Overall diagnostic performance of various diagnostic approaches

Index	Sensitivity % (95% CI)	Specificity % (95% CI)	Accuracy % (95% CI)	AUC % (95% CI)	*P*^#^ value	P^*^ value
ChatGPT-4o	**78.0** **(62.4–89.4)**	36.0 (25.2–47.9)	50.9 (41.4–60.3)	57.0 (48.6–65.5)	0.008	0.393
Claude-3-Opus	70.7 (54.5–83.9)	33.3 (22.9–45.2)	46.6 (37.2–56.0)	52.0 (43.2–60.9)	0.002	/
Junior radiologist	63.4 (46.9–77.9)	**81.3** **(70.7–89.4)**	**75.0** **(66.1–82.6)**	**72.4** **(63.7–81.1)**	/	/

*P*^*#*^ value indicates the comparison of AUCs between ChatGPT-4o, Claude-3-Opus, and Junior radiologist. P^*^ value indicates the comparison of AUCs between ChatGPT-4o and Claude-3-Opus. The bold value signifies the highest diagnostic performance in this metric

*AUC* area under the curve, *CI* confidence interval

### Unnecessary biopsy rates comparison: LLMs vs. junior radiologist

The rates of unnecessary biopsies varied across the diagnostic approaches (Table 4, Fig. 4). ChatGPT-4o recommended biopsies for 80 nodules, of which 48 were unnecessary, resulting in an unnecessary biopsy rate of 41.4% (48/116). Claude 3-Opus recommended biopsies for 79 nodules, with 50 being unnecessary, yielding an unnecessary biopsy rate of 43.1% (50/116). In contrast, the junior radiologist recommended biopsies for 40 nodules, with 14 being unnecessary, translating to a lower unnecessary biopsy rate of 12.1% (14/116).

**Table 4:** Comparison of unnecessary biopsy rate between various diagnostic approaches

Index	No. of recommended biopsy nodules	No. of malignant nodules^a^	No. of benign nodules^a^	Unnecessary biopsy rate, %^b^
ChatGPT-4o	80	32 (40.0)	48 (60.0)	41.4 (48/116)
Claude-3-Opus	79	29 (36.7)	50 (63.3)	43.1 (50/116)
Junior radiologist	40	26 (65.0)	14 (35.0)	**12.1 (14/116)**

^a^Data are presented as *n* (%). ^b^Data are presented as percentage (numerical structure ratio). The bold value signifies the highest diagnostic performance in this metric

## Discussion

In this study, we explored the application of LLMs for the analysis of ultrasound images to address critical medical diagnostic challenges. Specifically, we evaluated the performance of two state-of-the-art LLMs, ChatGPT-4o and Claude 3-Opus, in classifying thyroid nodules based on ultrasound imaging. To the best of our knowledge, this research represents the first study of utilizing LLMs for the direct analysis of thyroid nodule ultrasound images to distinguish between benign and malignant nodules. Our results demonstrate that while these LLMs exhibit some potential, their performance in differentiating benign from malignant thyroid nodules is limited, falling significantly short of the diagnostic accuracy achieved by a junior radiologist. Importantly, it must be stressed that neither the LLMs nor the radiologist achieved strong performance in the evaluated tasks. In fact, both the models and the radiologist performed below acceptable clinical thresholds, indicating that improvements are needed in both automated and human-driven diagnostic processes for this task.

In the present study, thyroid nodule ultrasound images were input directly into LLMs to assess their benign or malignant nature, with histopathological findings serving as the gold standard for comparison. The TI-RADS classification system was not utilized for risk stratification. Although various TI-RADS systems, such as ACR, EU, ATA, and Korea, are widely used, a universally accepted TI-RADS classification has not yet been established across all regions [14–17]. Each TI-RADS system differs in how malignancy risk is categorized, and no consistent cutoff exists to reliably separate benign from malignant nodules across these systems. Additionally, significant differences in sensitivity and specificity are observed among the TI-RADS systems, making it challenging to harmonize diagnostic approaches and compare results across studies or regions [18, 19]. While TI-RADS is recognized as an essential tool for clinical decision-making, certain diagnostic models are increasingly being developed to directly predict the likelihood of malignancy from ultrasound images. Several commercially available computer-aided diagnosis (CAD) systems, which are based on large-scale image datasets and deep learning algorithms, have already been designed to input ultrasound images and classify nodules directly as benign or malignant without relying on intermediary TI-RADS scoring [20–22]. This direct assessment approach is becoming a central focus in both research and clinical applications, as it allows for more streamlined diagnostic processes and may reduce interobserver variability inherent in TI-RADS interpretation. Consequently, this study was designed to explore the feasibility of using LLMs to directly classify thyroid nodules as benign or malignant without relying on intermediary TI-RADS-based stratification. This approach aligns with the current trend in CAD software development, where the ultimate goal is to generate a binary benign-versus-malignant outcome, providing a practical and efficient diagnostic tool for clinicians. By bypassing the inconsistencies between different TI-RADS systems, this study investigated the potential of LLMs to serve as an adjunct or alternative to traditional classification systems, with a focus on improving diagnostic accuracy and operational efficiency. Nevertheless, it is acknowledged that future studies could benefit from comparing the performance of LLMs with specific TI-RADS classifications to further assess their utility within existing clinical frameworks.

Previous studies have explored the application of LLMs in thyroid nodule evaluation, though predominantly focusing on text-based data or the analysis of ultrasound reports. For instance, Wu et al. demonstrated that LLMs like ChatGPT-4.0 achieved high diagnostic accuracy when combined with image-to-text strategies to analyze ultrasound features and structured diagnostic data [23]. This study reported an AUC of 0.83, which outperforms the results observed in our study. Similarly, Wang et al. applied a “Chain of Thought” methodology to deconstruct the decision-making process in ChatGPT-4.0’s analysis of thyroid ultrasound reports, thereby improving both its interpretability and diagnostic utility [24]. The superior performance of LLMs in these studies can be attributed to their reliance on image-to-text approaches or structured text-based reports, which act as intermediaries by converting visual data into structured, text-based inputs that LLMs are inherently more adept at handling. LLMs are trained predominantly on textual data and excel in tasks involving language comprehension, logical reasoning, and structured report generation [25–27]. These methods allow LLMs to capitalize on their strengths in processing textual information, thereby bypassing the challenges of direct pixel-level image analysis [28].

In contrast, our study directly evaluated the performance of LLMs, specifically ChatGPT-4o and Claude 3-Opus, in analyzing ultrasound images of thyroid nodules. Despite the advanced natural language processing capabilities of these models, they exhibited poor concordance with pathological diagnoses, with *Kappa* values of 0.116 for ChatGPT-4o and 0.034 for Claude 3-Opus. This demonstrates that current LLM technologies struggle to capture the nuanced visual cues essential for accurate ultrasound-based diagnosis. Furthermore, both LLMs significantly underperformed in comparison to a junior radiologist, with notably lower AUC values (ChatGPT-4o vs. radiologist: 57.0% vs. 72.4%, *P* = 0.008; Claude 3-Opus vs. radiologist: 52.0% vs. 72.4%, *P* = 0.002). These results underscore the limitations of current LLMs in medical imaging tasks, particularly those that require precise differentiation of nodule characteristics. While ChatGPT-4o and Claude 3-Opus exhibit potential in data processing and decision support tasks, their performance in image-based diagnostics remains significantly inferior to that of human experts.

A key factor contributing to the underperformance of LLMs in medical imaging is the misalignment between their design and the demands of image analysis. Trained primarily on textual data, LLMs excel in natural language understanding and reasoning but lack the capacity to process and analyze complex visual information, particularly at the pixel level [29, 30]. This mismatch is especially evident in the interpretation of thyroid ultrasound images, where subtle grayscale contrasts, edge delineations, and textural variations often signal malignancy. Such features are challenging for models trained on text-based inputs. Additionally, the training data for LLMs often lacks the depth and diversity needed to capture pathology-specific anatomical details visible in ultrasound images. LLMs are not optimized to process pixel-level information or comprehend spatial relationships, both of which are essential for accurate medical image interpretation.

It is essential to recognize that current LLM architectures do not incorporate the capability to perform medical image-based tasks that demand the interpretation of spatial, visual, and contextual patterns. These tasks require specialized models, such as those trained on multimodal data that integrates both text and images, or deep learning models specifically designed for medical image analysis. Future research should focus on developing and training models tailored for medical imaging tasks, incorporating both textual and visual data to create architectures capable of more effectively interpreting complex medical image features [31]. Such advancements could significantly enhance the diagnostic performance of LLMs in medical imaging and enable more accurate, automated image interpretation. However, before these models can be widely adopted, extensive validation studies are required to ensure their reliability and safety in clinical environments. Addressing these research and validation gaps is critical for transforming LLMs from a promising tool into a practical solution for medical imaging.

This study has several limitations that should be considered. The sample size was relatively small; future studies should include a larger cohort to further validate the diagnostic performance of LLMs. Additionally, the study did not account for the potential influence of image quality and variability in ultrasound equipment and techniques, which could affect the performance of the LLMs.

## Conclusions

This study demonstrates the potential of LLMs, specifically ChatGPT-4o and Claude 3-Opus, in classifying thyroid nodules based on ultrasound images. However, their diagnostic performance is currently limited and falls short of that achieved by a junior radiologist. This underscores the inherent limitations of these models in medical imaging tasks and highlights the cautious stance of medical professionals regarding their application in clinical settings. To effectively integrate LLMs into medical imaging diagnostic workflows, future efforts should focus on optimizing LLM architectures for medical imaging, expanding training datasets, and improving their diagnostic capabilities and reliability in clinical practice.

## Data Availability

The data presented in this study are available from the corresponding author upon reasonable request. Data is not publicly available due to privacy or ethical concerns.
